# Characterization of the complete chloroplast genome of a Chinese endangered species *Cymbidium mannii*

**DOI:** 10.1080/23802359.2020.1810159

**Published:** 2020-08-25

**Authors:** Yuying Wang, Longjie Cheng, Yiran Zhao, Yefang Li, Fengmei He, Zhilin Li

**Affiliations:** College of Horticulture and Landscape, Yunnan Agricultural University, Kunming, China

**Keywords:** *Cymbidium mannii*, chloroplast genome, endangered species, phylogenetic analysis

## Abstract

*Cymbidium mannii* is an endangered species belonging to the first ranking in protection category in China with important ornamental value and breeding value. This study used Illumina high-throughput sequencing technologies to sequence and analyze the complete chloroplast genome of *C. mannii.* The genome features of *C. mannii* and the phylogenetic relationships among Orchidaceae species were reported and established. The complete chloroplast genome is 152,544 bp in length, consisting of a pair of inverse duplication regions 25,357 bp, a large single-copy region 86,014 bp and a small single-copy region 15,816 bp. The entire genome contains 74 mRNA genes, 30 tRNA genes and 4 rRNA genes. The phylogenetic tree of 23 Orchidaceae species revealed *C. mannii* is more closely related to *Cymbidium aloifolium*.

*Cymbidium mannii* (Orchidaceae) is a shrub that is widely distributed in tropical and subtropical areas of southeast Asia and southern provinces of China and attached to forest tree trunks at altitudes of 100–1600 m (Liu et al. 2009). *Cymbidium mannii* has been listed as a ClassIprotected plant in the China Rare and Endangered Plants List (http://www.iplant.cn/rep/protlist). It has high ornamental value and beautiful flowers, the sepals and petals pale to creamy yellow. The length of inflorescence with droop or bend downward is 17–28 cm with 10–20 or more flowers. The flowering period is longer and ranges from February to April (Sheng et al. 2013). Due to its high value and rarity, the wild *C. mannii* is threatened by over-collection from its natural habitat (Wei et al. 2012).

The complete chloroplast genome sequences of *C. mannii* was obtained (GenBank Accession No. MT576626). The genome sequences and features are significant to study the phylogenetic relationship of *C. mannii* and helpful for the in-depth study of the chloroplast. Besides, it plays an essential role in the diversity research of genetic resources of this plant. Specimens of *C. mannii* were gathered from the Flower Research Institute of College of Horticulture and Landscape, Yunnan Agricultural University, Kunming, Yunnan Province, China (25°07′43″ N, 102°44′54″ E), and specimens were deposited in the Herbarium of Kunming Institute of Botany of CAS (specimen code: CY001). A modified CTAB method (Doyle and Doyle 1987) was used to extract the entire chloroplast DNA of *C. mannii* from fresh mesophyll tissue.

Sequencing was performed using the Illumina NovaSeq in GENOSEQ Technologies Limited Company (Wuhan, China). The raw reads and clean reads were obtained and then were assembled by NovoPlasty (Dierckxsens et al. 2017). The assembled contigs were compared with the chloroplast genomes of the closely related species through the use of blastn (version: BLAST 2.2.30+; parameter: -evalue 1e-5). Then the contigs were checked, selected, adjusted to get the final data. The chloroplast genome was annotated and mapped by using GeSeq (Tillich et al. 2017).

The length of complete chloroplast genome of *C. mannii* is 152,544 bp. The genome presented a characteristic quadripartite circular structure which included one pair of inverted repeat regions (IRs, 25,357 bp), one large single-copy region (LSC, 86,014 bp) and one small single-copy region (SSC, 15,816 bp). Besides, the complete genome contains 74 messenger RNA genes, 30 transfer RNA genes and 4 ribosomal RNA genes. The overall GC content of *C. mannii* chloroplast genome is 36.92%. Moreover, the GC content of IR regions (43.41%) is higher than the LSC region (34.41%) and the SSC region (29.81%).

To study the phylogenetic relationship of *C. mannii*, a phylogenetic tree was constructed by using 20 complete chloroplast genomes of *Cymbidium* species and three Orchidaceae species were selected as an outgroup. All the sequences were downloaded from NCBI GenBank. All sequences of species were aligned by the online program MAFFT version 7 and MEGA v7.0 was used to build the maximum-likelihood phylogenetic tree with 1000 rapid bootstrap replicates (Kumar et al. 2016). The phylogenetic tree analysis indicated that *C. mannii* was closely related to *Cymbidium aloifolium* (Figure 1).

**Figure 1.**
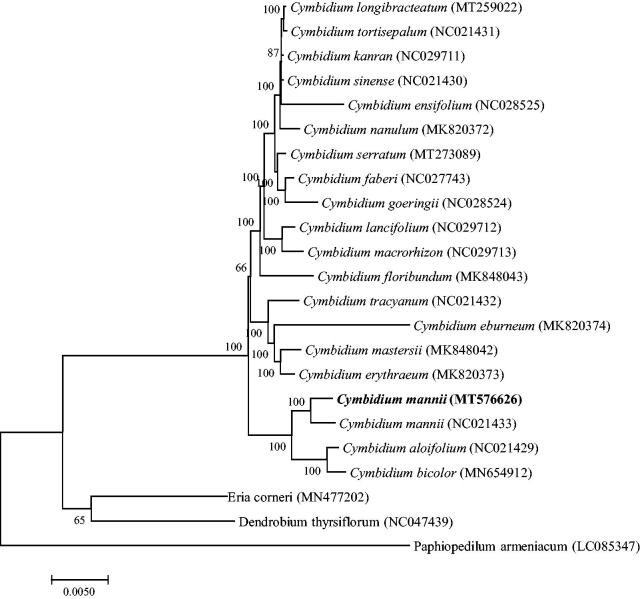
A Phylogenetic tree based on 23 complete chloroplast genome sequences of Orchidaceae species using the Maximum Likelihood (ML) analysis by MEGA v7.0. Bootstrap support values are indicated in each node.

## Data Availability

The data that support the findings of this study are openly available in GenBank of NCBI at https://www.ncbi.nlm.nih.gov, reference number MT576626.
